# Supplementary material for: Differences in suicide acceptability by farming-related occupation, demographic, and religiosity factors, general social survey 2000–2022
Source: PLOS Ment Health. 2024 Dec 4;1(7):e0000073. doi: 10.1371/journal.pmen.0000073 (PMC12798634; doi:10.1371/journal.pmen.0000073)
Supplement: Attachment — Submitted filename: Response to Reviewers.docx [file pmen.0000073.s001.docx]

Differences in Suicide Beliefs by Farming-Related Occupation, Demographic, and Religiosity Factors, General Social Survey 2000-2022

Response to reviewers

Reviewers' comments:

Reviewer #1: In this study, the authors utilize data from the Cross-sectional General Social Survey (GSS) collected between 2000 and 2022 to investigate attitudes toward suicide among individuals working in agriculture. Specifically, the authors explore the relationship between the attitudes toward suicide in this population, demographic factors, and spirituality.

Methodologically, the authors employ bivariate categorical statistical tests and multivariable logistic regression for their analysis. The primary hypothesis posited by the authors suggests that suicide is more accepted among those employed in agricultural occupations compared to the general population of the USA. However, the findings presented in the study do not support this hypothesis, adding complexity to the research.

This study contributes to the broader context of developing tailored approaches to address suicide prevention among farmworkers, a recognized at-risk group. The unexpected results prompt further inquiries into the underlying factors contributing to suicidal ideations within the agricultural population. Questions arise regarding the identification of specific risk and protective factors unique to this demographic and how this knowledge can inform improved suicide prevention strategies for agricultural workers.

While the authors present their findings effectively, there are minor issues that could enhance the clarity and rigor of the paper. Firstly, it is crucial to ensure that the sample of 167 agricultural worker respondents is nationally representative of the broader agricultural workforce in the United States. Additionally, providing more detailed information in the methodology section regarding the specific statistical methods employed would improve transparency and reproducibility. Lastly, sharing the psychometric properties of the GSS items used to measure suicide acceptability would aid in the interpretation of the findings, offering insights into the reliability of the measurement tools utilized.

I believe this paper makes a valuable contribution to the field by investigating the attitudes toward suicide among agricultural workers and advocating for tailored prevention strategies. With some minor revisions addressing the outlined issues, I recommend this paper for publication.

**Response: We thank the reviewer for their time and detailed review. We have carefully considered the reviewer’s constructive comments, and our responses to each are explained in greater detail below.**

Minor issues:

1). The aim of the authors is to create a nationally representative study. The GSS dataset used by the authors is nationally representative of its 18,191 respondents, however it is not clear to me that the 167 respondents of the agricultural worker subgroup is a nationally representative of the agricultural workers of the United States.

**Response: Because the GSS surveys a representative sample of U.S. respondents, the estimates we generated should also be nationally representative. However, we acknowledge the sample size of the agricultural workers is relatively small, contributing to instability of estimates. Thus, in response to the reviewer’s comment, we added text to the limitations to explain this point and to compare our sample with previous studies to assess representativeness (lines 286-297).**

2) The methodology section of the paper could benefit from more details. For example, the authors write “Bivariate categorical statistical tests (e.g., chi-square) of demographic variables and suicide acceptability were used to examine differences between farm/ranch operators, farm/ranch workers, and the general population.” (128-130), I would like to know which methods were used in which cases.

**Response: We have edited the methodology section to more clearly indicate the statistical tests that were conducted for each type of variable (lines 157-161).**

3) Sharing the psychometric properties of the GSS items that were used to measure suicide acceptability would aid the readers of this paper to interpret its findings. The authors support the use of these items by showing that they have been used in previous research, however we do not know whether the four items are reliable or not in measuring the acceptability of suicide.

**Response: The reliability of the GSS suicide acceptability scale is discussed further (lines 124-130) in the methods, along with a citation to previous research that also reports on the psychometrics of the items. We also examined the Cronbach’s alpha of the GSS suicide acceptability scale in the analytic sample used in the present analysis, which was .77 and considered reliable.**

Reviewer #2: It is one of the most crucial issues that our society will confront in the not-too-distant future, and I applaud the writers for going for it. When we're going through tough times, and I think this is a way to find meaning in life for the future when a human thinks others want to kill us (diseases, economic pressure or life course events).

1. The authors should present broad definitions in the opening part before digging into specific themes for instructional reasons. These definitions should cover factors such as occupation, demographics, and religiosity. There are a lot of people who are under the incorrect impression that these aspects are not significant and should simply be regarded control variables. Researchers are able to better comprehend the many influences and viewpoints that may have an effect on the results of the study by gaining a better understanding of these aspects, which are crucial since they give context. The writers are able to conduct a more thorough analysis of the data and arrive at results that are more accurate and that accurately reflect the diversity of the population that is being examined if they take into account aspects such as occupation, demographics, and religiosity.

**Response: We expanded the introduction to include definitions and more details about the significance of occupation, demographics, and religiosity (lines 6-13, 50-51, and 68-78).**

2. In the discussion section, there is potential for improvement regarding the issues of occupation, demographics, and religiosity as they pertain to the society of the United States of America. From the perspective of readers in the United States and all around the world, the conclusion needs to offer a mix of general and specific ideas that will assist in advancing this agenda in the course of future research. The factors of occupation, demographics, and religious affiliation all play a significant part in the formation of society dynamics and individual experiences in the framework of the United States. For example, one's occupation not only impacts their economic standing but also affects their access to resources, opportunities, and social networks. Age, ethnicity, and gender are all examples of demographic factors that lead to inequalities in education, healthcare, and representation. On the other hand, religiosity has an effect on social norms, values, and beliefs, which in turn has an effect on social cohesiveness and political ideologies. In order to achieve a full understanding of the society in the United States and to direct future study toward addressing issues that are relevant, it would be beneficial to recognize and analyze these elements in the discussion and conclusion sections. Furthermore, the interaction between work, demography, and religious affiliation can also be a contributor to social problems that bring individuals to the verge of suicide and cause them to consider ending their own lives. One example of a toxic environment that can contribute to feelings of hopelessness and despair is when a number of circumstances come together. These elements include unemployment, low socioeconomic position, discrimination based on demographics, and rigid religious beliefs. It is essential to have a solid understanding of these links in order to design effective interventions and support systems that can help prevent tragedies of this nature and improve mental well-being.

**Response: We appreciate the reviewer highlighting these topics. In response, we edited the discussion to elaborate on the importance of occupation, demographics, and religious affiliation with the outcome (lines 305-320, 349).**
