# Supplementary material for: Differences in suicide acceptability by farming-related occupation, demographic, and religiosity factors, general social survey 2000–2022
Source: PLOS Ment Health. 2024 Dec 4;1(7):e0000073. doi: 10.1371/journal.pmen.0000073 (PMC12798634; doi:10.1371/journal.pmen.0000073)
Supplement: Attachment — Submitted filename: Response to ReviewersR2.docx [file pmen.0000073.s002.docx]

Differences in Suicide Acceptability by Farming-Related Occupation, Demographic, and Religiosity Factors, General Social Survey 2000-2022

Response to Reviewer #3

**Response: We thank the reviewer for their time and detailed review. We have carefully considered the reviewer’s constructive comments, and our responses to each are explained in greater detail below.**

Reviewers' comments:

1. Introduction: It is recommended that suicide beliefs should be discussed or defined and how this encompasses suicide acceptability or endorsement. Since the construct of suicide beliefs is what is in the title, kindly establish the scope and de/limitations. Are the terminologies suicide beliefs and suicide acceptability used interchangeably?

**Response:** In the introduction, we clarified the construct of suicide beliefs to encompass suicide acceptability. We also changed the article title to clarify that we are looking specifically at suicide acceptability beliefs, which are one type of suicide beliefs. These are not interchangeable terms.

2. Methods: Is the sample size of 167 people who worked in farming-/ranching-related representative of the population?

**Response**: **The GSS sampling is representative of the U.S. population. In the sample, 1% of respondents identified as being in farming-/ranching populations, which is the same as the U.S. population. A new citation has been added for that, and this point is discussed further in the Discussion.** **We have added more detail about the sampling process for the GSS questionnaire as well.**

3. If the four items in General Social Surveys (GSS) “measure” suicide acceptability, do these four items “measure” suicide beliefs? Kindly use the term that is directly measured by your tool/questionnaire. Your scope and de/limitations may guide you to clearly phrase the title as well.

**Response: We have changed the title to more accurately describe suicide acceptability, which is being measured by the four items on the survey, and is a component of suicide beliefs. Throughout the paper we edited the language to consistently refer to suicide acceptability because it was the specific type of belief assessed.**

4. Abstract Results: “Among 18,191 respondents to the GSS, 167 people worked in farming/ranching roles, including 74 farmers/ranch operators and 93 farm/ranch workers.” This statement is more appropriate in the Methods than in the Results.

**Response**: **This has been moved to the Methods.**

5. “The analysis focused on four items asking respondents about suicide acceptability, defined as rationalizing suicide as a viable option when faced with adversity (52). Studies have found links between higher suicide acceptability/endorsement and suicide rates (37, 53), and studies with GSS data have measured this concept in various U.S. populations, including those facing financial and legal hardship (36), veterans 115 (54), sexual minorities (55), Black and African-116 Americans (56), and population-wide (35).” These statements in the Methods part made this confusing for me. I wish to read the details of the Methods only. These statements may be included in the Introduction part.

**Response: This has been moved to the Introduction.**

6. Methods: Kindly focus on describing the questionnaire

**Response**: **We have elaborated on the questionnaire in the Methods section.**
